# Primary hypothyroidism with an episode of ventricular tachycardia in a patient with COVID-19: A case report

**DOI:** 10.1097/MD.0000000000029243

**Published:** 2022-06-24

**Authors:** Pin-Hsu Liao, Yu-Cheng Cheng, Po-Yu Liu, I-Te Lee

**Affiliations:** a Division of Endocrinology and Metabolism, Department of Internal Medicine, Taichung Veterans General Hospital, Taichung City, Taiwan; b School of Medicine, Chung Shan Medical University, Taichung City, Taiwan; c School of Medicine, National Yang Ming Chiao Tung University, Taipei, Taiwan; d Division of Infectious Diseases, Department of Internal Medicine, Taichung Veterans General Hospital, Taichung City, Taiwan.

**Keywords:** coronavirus disease 2019, hypothyroidism, levothyroxine, ventricular tachycardia

## Abstract

**Rationale:**

Coronavirus disease 2019 (COVID-19) is a systemic disease with major clinical manifestations in the respiratory system. However, thyroid involvement has also been reported. We present a case of hypothyroidism with ventricular tachycardia following diagnosis with COVID-19.

**Patient concerns:**

A 77-year-old man was admitted to the isolation ward due to COVID-19. After respiratory support and medical treatment, the patient was successfully weaned off the ventilator. However, an episode of short-run ventricular tachycardia was detected, and primary hypothyroidism was also diagnosed.

**Diagnosis:**

Ventricular tachycardia was detected by electrocardiography.

**Interventions:**

Intravenous amiodarone administration and oral levothyroxine replacement.

**Outcomes:**

No arrhythmia detected following treatment.

**Lessons:**

Awareness of the association between hypothyroidism and COVID-19 is important in preventing possible complications.

## 1 Introduction

Coronavirus disease 2019 (COVID-19), caused by severe acute respiratory syndrome coronavirus 2 (SARS-CoV-2), is a systemic disease.^[1]^ Although the majority of clinical presentations seen in COVID-19 are characterized by respiratory system manifestations, thyroid involvement has also been reported.^[2,3]^ This case report deals with the diagnosis of hypothyroidism following an episode of ventricular tachycardia in a patient with COVID-19. Awareness of the association between hypothyroidism and COVID-19 is important in preventing possible complications.

## 2 Case report

A 77-year-old man with a history of coronary artery disease underwent percutaneous coronary intervention >10 years ago. The patient had remained in stable cardiovascular condition since then, with medical treatment consisting of aspirin, anti-hypertensive drugs, and statins. After that, he had no history of hospitalization until May 20, 2021, when he was diagnosed with COVID-19.

**Figure 1:**
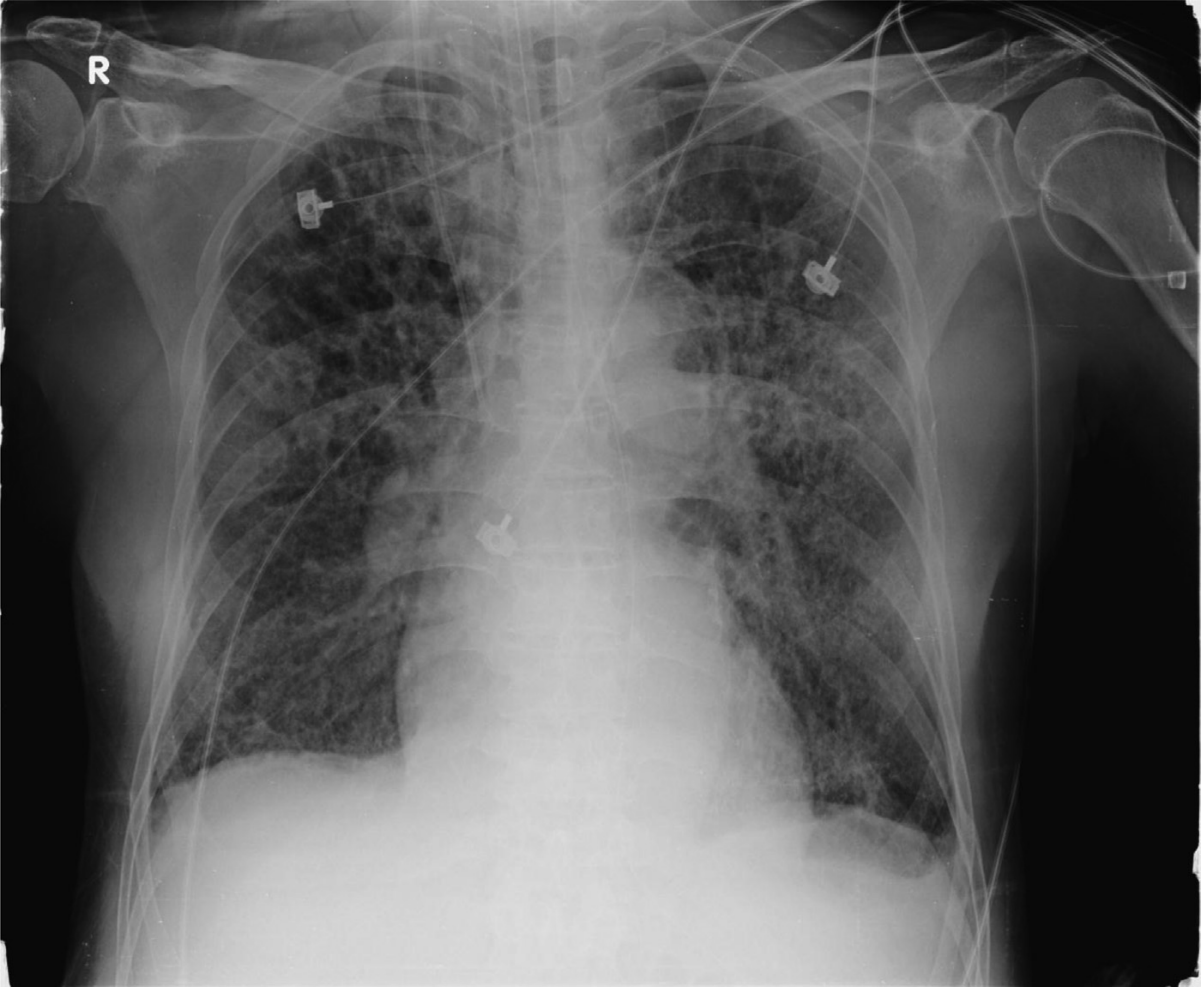
Typical radiological findings seen on a chest radiograph of the patient following endotracheal intubation for acute respiratory distress syndrome.

His initial presentation was a dry cough, observed on May 17, 2021, which was followed by intermittent diarrhea. He was admitted to the isolation ward because a reverse transcription polymerase chain reaction detected the presence of SARS-CoV-2 RNA from his nasopharyngeal swab, on May 20, 2021. Due to his progressive pneumonia, he was transferred to the intensive care unit at Taichung Veterans General Hospital, and endotracheal intubation with mechanical ventilation was performed due to the patient suffering from acute respiratory distress syndrome. The relevant radiograph is shown in Fig. 1. The arterial gas analysis showed a pH of 7.236, arterial partial pressure of oxygen of 72.2 mm Hg, and arterial oxygen saturation of 90.4%, while the fraction of oxygen was 50% support in volume control mode with a positive end-expiratory pressure of 10 cmH_2_O. In addition to respiratory support, the patient received medical treatment, including initial remdesivir (200 mg) and tocilizumab (600 mg), followed by levofloxacin (750 mg), remdesivir (100 mg), and dexamethasone (6 mg) daily.

**Figure 2:**
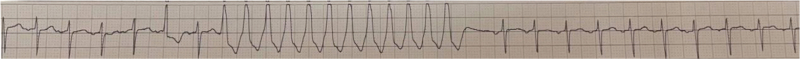
The short-run ventricular tachycardia rhythm detected by electrocardiography monitoring.

After his condition improved, the patient was successfully weaned off the ventilator and underwent extubation on the morning of June 2, 2021. However, 10 hours later, a 3-second episode of short-run ventricular tachycardia was detected by electrocardiography monitoring (Fig. 2). A panel of relevant investigations was performed immediately after the episode of arrhythmia. The serum concentrations of sodium, potassium, calcium, magnesium, and phosphates were all within their normal ranges. There was no acute myocardial ischemic change based on 12-lead electrocardiograms. Normal serum levels of creatine kinase (CK) of 158 U/L (normal range: 10–160 U/L) and creatine kinase myocardial band of 7 U/L (normal range: <16 U/L) were observed. The low-density lipoprotein cholesterol level was 1.4 mmol/L. However, a low serum free thyroxine level of 0.53 ng/dL (normal range: 0.70–1.48 ng/dL), a low triiodothyronine level of 41 ng/dL (normal range: 64–152 ng/dL), and an elevated thyroid-stimulating hormone level of 118.25 μIU/mL (normal range: 0.35–4.94 μIU/mL) were detected. A series of arterial blood gas data are displayed in Table 1.

**Table 1:** A series of arterial blood gas analyses.

Clinical events	Date	pH	PaCO_2_, mmHg	PaO_2_, mmHg	SO_2_ (%)	BE, mmol/L	HCO_3_, mEq/L
Normal range		7.35–7.45	35–45	80–100	≥95	–2–2	22–26
Ventilator support	May 26	7.236	59.8	72.2	90.4	–3.8	24.8
Before extubation	June 02	7.459	42.6	72.9	95.6	5.2	29.6
After extubation^∗^	June 02	7.474	38.2	81.0	96.8	3.8	27.5
After VT occurrence	June 03	7.520	36.2	66.3	95.2	6.0	28.9

BE = base excess, HCO_3_ = bicarbonate, PaCO_2_ = arterial partial pressure of carbon dioxide, PaO_2_ = arterial partial pressure of oxygen, SO_2_ = arterial oxygen saturation, VT = ventricular tachycardia.

∗ Before VT occurrence.

After intravenous amiodarone (150 mg) bolus injection, followed by a continuous drip (450 mg) for 8 hours, and another continuous drip (450 mg) for 16 hours, ventricular tachycardia was no longer detected on the electrocardiography monitor. After consultation with an endocrinologist, levothyroxine replacement was suggested, beginning with an initial dose of 50 μg per day, under the impression of primary hypothyroidism. Further investigations showed an antithyroid peroxidase antibody level of 671.55 IU/mL (normal range <5.61 IU/mL), an antithyroglobulin antibody level of 776.26 IU/mL (normal range <4.11 IU/mL), a CK level of 275 U/L, and a creatine kinase myocardial band level of 5 U/L. On June 23, 2021, the patient's thyroid-stimulating hormone level had reduced to 59.594 μIU/mL, and the free thyroxine level was 0.85 ng/dL. The patient was discharged in relatively stable condition on June 30, 2021. No arrhythmia was detected during outpatient visits. The results of the patient's thyroid function tests are shown in Table 2. Echocardiography revealed normal systolic wall motion of the left ventricle, with an ejection fraction of 55% on July 30, 2021.

**Table 2:** Thyroid function test results and date of reports.

	Reference range	June 3	June 18	June 23	August 4
TSH, μIU/mL	(0.35–4.94)	118.25	83.71	59.59	19.38
Free T4, ng/dL	(0.70–1.48)	0.53	0.63	0.85	1.01
T3, ng/dL	(64–152)	41.12			114.89
Anti-TPO antibody, IU/mL	(<5.61)	671.55			
Anti-TG antibody, IU/mL	(<4.41)	776.26			

T3 = triiodothyronine, T4 = thyroxine, TG = thyroglobulin, TPO = thyroid peroxidase, TSH = thyroid stimulating hormone.

## 3 Discussion

It has been reported that hypothyroidism is associated with ventricular tachycardia.^[4]^ Nesher and Zion^[5]^ documented the repeated occurrences of ventricular tachycardia in a woman with autoimmune-associated hypothyroidism and reviewed 4 other female patients with established hypothyroidism and ventricular tachycardia. Notably, in our case, the episode of ventricular tachycardia and hypothyroidism were both diagnosed during the patient's hospitalization for COVID-19.

It is evident that COVID-19 can induce cytokine storms and destroy the hormone-producing ability of the thyroid gland.^[6]^ The proposed mechanism for thyroid dysfunction in COVID-19 involves angiotensin-converting enzyme-2 (ACE-2) present on the thyroid gland.^[7]^ Infiltration of the thyroid gland through these ACE-2 activates cytokines that destroy follicular cells, and results in the classical clinical picture seen in hypothyroidism as well as the presence of anti-thyroid peroxidase antibodies.^[6,7]^ Thyroid hormones play an important role in the cardiovascular system.^[8]^ Hypothyroidism is associated with cardiac structure changes and conduction abnormalities.^[9,10]^

In addition to the thyroid gland, cardiomyocytes and cardiac pericytes also express ACE-2, which may act as a target for SARS-CoV-2.^[11,12]^ The high inflammatory burden caused by cytokine release in COVID-19 has the potential to induce acute myocardial injury and arrhythmia.^[13]^ Therefore, prompt diagnosis and management of cardiac arrhythmias are important considerations in clinical practice, when dealing with COVID-19 infection.^[14]^ The presence of hypothyroidism might worsen the prognosis in such patients after they are diagnosed with COVID-19.^[15–17]^

The ACE-2 is an important entry receptor in host cells and is involved in multiple pathogenic pathways. ACE-2 expression levels are relatively higher in the thyroid gland and heart, than in the lung.^[18]^ Approximately 15% of adults with COVID-19 infection have abnormal thyroid function, and circulating triiodothyronine levels are associated with adverse COVID-19 outcomes.^[19,20]^ Therefore, physicians should be aware of the possibility of thyroid dysfunction in patients with COVID-19. In the present case, his condition was stable following treatment with amiodarone, and levothyroxine replacement. Physicians should also closely monitor thyroid function since amiodarone itself has the potential to induce thyroid dysfunction.^[21]^ Steps should also be taken to avert an adrenal crisis during the withdrawal of steroids after thyroxine replacement.^[22]^

Certain other factors might be associated with ventricular arrhythmia. Acidosis and electrolyte imbalance can potentially induce ventricular arrhythmia.^[23,24]^ Several medications used in the treatment of COVID-19 also have arrhythmogenic risks,^[25–27]^ including hydroxychloroquine which was not administrated in the case we presented. The episode of ventricular tachycardia occurred during a relatively stable period, and respiratory acidosis had subsided around this period. The serum concentrations of sodium, potassium, calcium, magnesium, and phosphates were not abnormal upon assessment after the episode of ventricular tachycardia. However, there are several limitations to this case report. Firstly, we cannot confirm the cause per se of the ventricular tachycardia, since both hypothyroidism and COVID-19 have been known to precipitate ventricular arrhythmia. Secondly, we cannot establish the causation between COVID-19 and hypothyroidism, since there are no data on thyroid function before the patient was diagnosed with COVID-19. Finally, we cannot speculate whether there would have been recurrences of the ventricular arrhythmia in case thyroxine replacement had not been initiated.

## 4 Conclusion

We reported a patient diagnosed hypothyroidism following an episode of ventricular tachycardia after he was admitted with a diagnosis of COVID-19. Based on our observation, we wish to emphasize the need for increased awareness regarding the possibility of this association with earlier diagnoses and to propose an appropriate treatment.

## Acknowledgments

The authors thank the Department of Critical Care Medicine of Taichung Veterans General Hospital for their support.

## Author contributions

**Conceptualization:** Pin-Hsu Liao, Yu-Cheng Cheng, Po-Yu Liu, I-Te Lee.

**Data curation:** Yu-Cheng Cheng, I-Te Lee.

**Formal analysis:** Pin-Hsu Liao, I-Te Lee.

**Investigation:** Pin-Hsu Liao, Yu-Cheng Cheng, Po-Yu Liu, I-Te Lee.

**Methodology:** Pin-Hsu Liao, I-Te Lee.

**Project administration:** I-Te Lee.

**Resources:** I-Te Lee.

**Software:** Pin-Hsu Liao.

**Supervision:** Po-Yu Liu, I-Te Lee.

**Validation**: Po-Yu Liu, I-Te Lee.

**Visualization:** Pin-Hsu Liao, I-Te Lee.

**Writing – original draft:** Pin-Hsu Liao.

**Writing – review & editing:** Po-Yu Liu, I-Te Lee.
